# Exploration of COVID-19 Pandemic Prevention Behaviors among Healthcare Workers

**DOI:** 10.3390/healthcare11020153

**Published:** 2023-01-04

**Authors:** Hui-Ting Huang, Chung-Hung Tsai, Chia-Fen Wang, Tzu-Chao Chien, Shu-Hao Chang

**Affiliations:** 1 Taiwan Adventist Hospital, Taipei 10556, Taiwan; 2 Department of Information Technology and Management, Tzu Chi University of Science and Technology, Hualien 970302, Taiwan; 3 Science & Technology Policy Research and Information Center, National Applied Research Laboratories, Taipei 10636, Taiwan

**Keywords:** COVID-19, workplace safety climate, health belief model, pandemic prevention behaviors

## Abstract

Since the outbreak of COVID-19, the pandemic has become an important topic of global public health. To reduce the rapid spread of the pandemic, compliance with preventive behaviors has become one of the important guidelines from the World Health Organization (WHO). Healthcare workers stand on the frontline for pandemic prevention, and preventive behaviors are essential measures to protect their health and safety. The purpose of this study was to propose an integrative model that explained and predicted COVID-19 preventive behaviors among healthcare workers. The study integrated workplace safety climate and the health belief model (HBM) to verify the impact of workplace safety climate and health belief factors on the safety attitude, safety compliance, and safety satisfaction of healthcare workers performing COVID-19 pandemic prevention behaviors. A cross-sectional study was conducted from March to August 2021 with a self-administered online questionnaire. The sample of the study was drawn from healthcare workers of a famous medical institution in Taipei City as research subjects. After collecting 273 valid questionnaires and verifying them through the analysis of structural equation modeling (SEM), the findings revealed that workplace safety climate had an impact on health belief factors, and then health belief factors had impacts on safety attitudes. In addition, safety attitude affected safety compliance, while safety compliance further affected safety satisfaction. The study showed that workplace safety climate can strengthen healthcare workers’ health beliefs and further affect their safety attitudes, safety compliance, and safety satisfaction. The study attempted to propose a model of healthcare workers’ pandemic prevention behaviors as a reference for medical facility administrators in real practice.

## 1. Introduction

COVID-19 broke out at the end of 2019, and the World Health Organization (WHO) announced it as a “Public Health Emergency of International Concern” at the end of January 2020. In May 2020, the WHO also published relevant measures for infection prevention and control. The Ministry of Health and Welfare in Taiwan immediately referred to the relevant guidelines from the WHO and published important guidance and teaching materials, such as strategies for stopping community spread and guidance on pandemic prevention behaviors. These approaches reduced the impact of the infectious disease on individuals and communities during the pandemic of COVID-19 before the public was vaccinated [1]. Since the global outbreak of COVID-19 in 2019, there have been 8,578,053 confirmed cases in Taiwan as well as the deaths of 14,890 people [2]. Based on its previous experience in handling the pandemic of SARS, Taiwan’s government immediately took rapid and effective measures for COVID-19 pandemic prevention [3]. With dedicated efforts for more than two years, the number of confirmed cases and deaths was significantly lower than in most countries by early 2021 [4]. Currently, the pandemic of COVID-19 in Taiwan is being successfully controlled and monitored. Most studies attribute the effectiveness of pandemic prevention in Taiwan to the successful implementation of preventive and control measures [5]. These measures to stop the spread of the virus include avoiding any travel to countries that are seriously affected, carrying out regular disinfection with sodium hypochlorite, washing hands regularly, wearing a mask, maintaining social distancing, and prohibiting public gatherings [4]. Healthcare workers are on the frontline of pandemic prevention, and it is essential to ensure their protection from infection with the virus in order to prevent the spread of the virus and enhance the capabilities of medical care [6]. Therefore, understanding the behaviors of healthcare workers, including their personal health beliefs and compliance with safety behaviors for pandemic prevention, is very important for the prevention of COVID-19. Accordingly, the WHO has confirmed that the most critical part of prevention plans is safety behaviors for pandemic prevention, and these behaviors are the most effective methods to suppress rapid virus transmission [7]. In particular, healthcare workers who stand on the frontline are the first checkpoint of pandemic prevention; their knowledge and behaviors towards pandemic prevention are vitally important. In this context, this study focuses on healthcare workers as subjects to discuss their pandemic prevention behaviors towards COVID-19.

The purpose of this study was to explore pandemic prevention behaviors towards COVID-19 among healthcare workers from the viewpoints of the health belief model (HBM) and workplace safety climate. The HBM was developed in the 1950s by social psychologists, Hochbaum and Rosenstock [8,9]. The model was originally developed to understand why patients failed to follow preventive behaviors that addressed personal or community health threats [10,11]. Later on, the HBM was widely used in different areas of public health, such as vaccination, medication adherence, and diabetic care [10], as well as in the explanation of pandemic prevention behaviors [12,13]. The HBM is a comprehensive model among numerous models of health behaviors. In addition, previous studies also demonstrated that the HBM is a useful model for predicting or interpreting infectious diseases such as COVID-19 [14]. One retrospective study recently pointed out that the HBM shows good predictability in preventive behaviors towards COVID-19 [15]. Based on this, this study used the HBM as the starting point to explore pandemic prevention behaviors towards COVID-19 among healthcare workers.

Moreover, this study incorporated workplace safety climate into the model. Pandemic prevention behaviors among healthcare workers are affected not only by personal health beliefs but also by the factors of the workplace [16]. Previous studies revealed that workplace safety climate is correlated with pandemic prevention behaviors among healthcare workers [17], and it affects the overall effectiveness and willingness of healthcare workers’ participation in pandemic prevention. The pandemic of COVID-19 brings new challenges to medical institutions in terms of management. Other than the traditional measures for the prevention of virus spread, strengthening safety protection awareness among healthcare workers and creating a safety climate in the medical institution are more important than in the past. Considering the impact of workplace safety climate on pandemic prevention behaviors, this study also included in its considerations.

Recently, studies have used concepts of the HBM or workplace safety climate to explore COVID-19 preventive behaviors. These studies have shown promising findings about the antecedents and outcomes of prevention but still have not provided a comprehensive model of COVID-19 pandemic preventive behaviors among healthcare workers. In addition, few studies have so far combined the important variables of antecedents, mediators, and outcomes to develop and examine a comprehensive framework of COVID-19 prevention in the healthcare context. The present study aimed to integrate the HBM, workplace safety climate, and other important variables (safety attitude, safety satisfaction, and safety compliance) into an integrative and multi-perspective model to promote the design of better intervention policies, procedures, programs, and practices in the future.

In response to the pandemic of COVID-19, as the frontliners of pandemic prevention, healthcare workers’ performance of pandemic prevention behaviors is an essential factor in stopping the spread of COVID-19 and maintaining the capacity of medical care. Therefore, this study used it as the research motivation to comprehensively identify factors that affect pandemic prevention behaviors among healthcare workers.

## 2. Literature Review and Hypotheses

### 2.1. Workplace Safety Climate

Zohar [18] argued that safety climate is a special pattern of organizational climate. The global COVID-19 pandemic shows the necessity of creating a safety climate in the workplace. Safety climate is the common perception among employees towards organizational safety policies, procedures, and practices [16] and will affect occupational safety [19]. That is, safety climate is the subjective evaluation of safety in the workplace by employees [20]. In terms of healthcare, it is an active and positive measurement of safety in the workplace and is mainly designed and reviewed from the perspectives of healthcare workers [21]. The recognition of workplace safety by employees will affect their presentation of ideal safety behaviors in the workplace. Therefore, a good workplace climate can predict better safety behaviors demonstrated by employees [16]. A recent study of medical institutions also pointed out that workplace safety climate can significantly predict preventive behaviors and safety satisfaction among employees [20].

As stated above, employees will evaluate the environment of their workplace, including the practice and process of safety in the organization, and reflect how safety is evaluated. Good workplace safety climate includes the perception of the importance of workplace safety training plans, the perception of the attitude of safety management, and the perception of the risk of the workplace in the organization [18]. There have been more and more relevant studies carried out in recent years; for example, the research conducted by McGhan et al. [22] verified a significant correlation between the perception and stress of the safety climate in the workplace, turnover intentions, and work satisfaction among healthcare workers. The study considered the importance of workplace safety climate for the welfare of healthcare workers. The study pointed out that medical organizations must consider workplace safety a priority to enhance the awareness of medical practitioners. Due to the high impact of workplace safety on the physical and mental states of healthcare workers, some studies have focused on the factors affecting safety climate: for example, employees might change their opinions towards the organizational environment because they suffer from diseases or injuries at work [23], and existing safety plans in the organization can shape its safety climate [18]. Thus, a lot of studies in the past have highlighted the importance of workplace safety climate and proposed empirical research related to safety climate [16,17,22]. The pandemic of COVID-19 at the moment affects safe and effective healthcare capabilities provided by healthcare workers. Establishing a good safety climate in medical care organizations is one of the main strategies to achieve patient safety and healthcare service quality improvement [24].

### 2.2. Health Belief Model (HBM)

The health belief model (HBM) tries to explain the preventive health behaviors practiced by the public, such as compliance with preventive behaviors towards COVID-19 [13]. The origin of the HBM was in theories of social psychology, especially expectancy-value orientation and field theory [25]. Expectancy-value orientation means that people carry out certain behaviors based on the outcome expectation and outcome value caused by their actions or lack thereof [26]. The field theory proposed by Lewin [25] argued that individuals exist in a living space consisting of the following several fields. Some fields are positively evaluated, which indicates a positive valence, while some are negatively evaluated, meaning a negative valence. In addition, there are also some relatively neutral fields. Illness is the field of negative valence, and it will force people to leave the field. The daily activities of people are attracted by positive forces and excluded by negative forces [9,27,28]. Therefore, behaviors are determined by the level of goal achievement resulting from the specific action as judged by people. Assuming that the goal is to avoid health hazards, they will predict whether a specific action is helpful in reducing health threats. The HBM is a theory related to health behaviors and can explain preventive behaviors and reactions to the disease by people. Meanwhile, the HBM provides a useful framework for health behavior investigation and identifies factors related to health beliefs [29]. In addition, so far, the development of health beliefs has been applied in many fields of preventive health behaviors and achieved good outcomes [30].

From the above, the HBM proposes the observation of personal beliefs in health behaviors from an individual’s point of view, such as personal preventive behaviors and attitudes towards an epidemic infectious disease [13,31] as well as self-health management [32]. The main concepts of the HBM include perceived susceptibility, perceived severity, perceived benefits, perceived barriers, and self-efficacy. According to the HBM, people’s specific beliefs, meaning the perceived susceptibility to and perceived severity of diseases as well as the perceived benefits of and perceived barriers to disease preventive behaviors, will affect people’s health behaviors [30]. Important concepts are described below.

First of all, the definition of perceived susceptibility is the personal perception of the subjective risk of being infected with a specific disease [33]. In this study, perceived susceptibility refers to people’s believed risk level of being infected with COVID-19 [30]. Previous studies pointed out a correlation between perceived susceptibility and workplace safety climate. A good organizational safety climate will reduce job insecurity [34]. For an individual, a sound safety policy can bring peace of mind to employees and reduce the risk of suffering from diseases or accidents [35]. In addition, Pandit et al. [36] also pointed out that a workplace with a more positive safety climate perception will demonstrate a high level of safety perception and reduce the risk of suffering from diseases. A study on healthcare workers conducted by Aram et al. [37] showed that those who rated the safety system in the workplace as good and who worked in a working environment that was regularly assessed had a lower perceived risk of being infected with COVID-19. Therefore, we proposed Hypothesis H1a of the study.

**Hypothesis 1a** **(H1a).** *Workplace safety climate will negatively affect* *perceived susceptibility.*

Perceived severity means personal perceptions and feelings towards suffering from a certain disease [38,39]. In this study, perceived severity means people believe that the outcome of being infected with COVID-19 is severe [30]. Previous studies revealed that the safety solutions, policies, and actual actions performed by medical institutions will enhance the perceived severity among employees and create a workplace with a more positive safety climate so that employees will present high levels of hazard identification and safety risk perception [36]. Workplace safety climate affects employees’ emotions and feelings of risk recognition [40] and affects personal feelings towards the potential outcome of the illness. Moreover, Ricci et al. [41] also indicated that the improvement of the safety climate will reduce the severity of accidents. Therefore, we proposed Hypothesis H1b of the study.

**Hypothesis 1b** **(H1b).** *Workplace safety climate will positively affect* *perceived severity.*

Perceived benefits are evaluations of the adopted actions to effectively reduce the threat of suffering from diseases and reflect personal thoughts or environmental perceptions on the measures improving the diseases [38]. In this study, perceived benefits mean people believe that COVID-19 preventive behaviors performed can reduce the risk or severity of the disease threat [30]. Research on the healthcare industry conducted by McFadden et al. [42] revealed a positive correlation between safety climate and continuous quality improvement in a hospital. In addition, safety climate is also directly related to the enhancement of patient safety. An empirical study conducted by Schwatka et al. [43] from the viewpoint of self-determination theory showed that the safety climate of an organization positively affects the safety motivation among employees, while safety motivation can further affect the behavior of participation. That is, organizations with good safety protection and improvement initiatives increase the perception of not being injured or infected with diseases among employees. The interpretation of the messages of the external environment by an individual affects the perception of perceived benefits, such as the level of preparation or recognition towards pandemic diseases among the public [44]. Therefore, when a workplace has good preparation and a good safety climate towards infectious disease prevention, employees will receive more perceived benefits. Therefore, we proposed Hypothesis H1c of the study.

**Hypothesis** **1c (H1c).** *Workplace safety climate will positively* *affect perceived benefits.*

Perceived barriers mean the potential costs during the process of performing pandemic prevention behaviors, as subjectively assessed by an individual, such as physical and mental devotion [13,39]. In this study, perceived barriers mean that people believe that performing COVID-19 preventive behaviors will be limited by psychosocial, physical, or financial factors [30]. Previous studies revealed that the costs of pandemic prevention can be reduced if the Disease Control Center can effectively deliver preventive initiatives related to infectious diseases to enterprise owners, such as encouraging employees who might be exposed to patients with COVID-19 to stay at home or providing relevant guidance to employees who show relevant symptoms [17]. Etafa et al. [45] pointed out that a failure to provide proper personal protective equipment to healthcare workers in hospitals, insufficient supportive medication, and the lack of proper ventilation equipment are barriers to COVID-19 pandemic prevention behaviors. A workplace with a good safety climate is one of the improvement factors for COVID-19 infection, prevention, and control. For employees, a clear safety policy and guidance provided by organizations will help to reduce barriers to employees’ participation in pandemic prevention behaviors. Therefore, we proposed Hypothesis H1d of the study.

**Hypothesis** **1d (H1d).** *Workplace safety climate will negatively* *affect perceived barriers.*

Previous studies have argued to include self-efficacy in the HBM [46]. In this study, self-efficacy means the level of confidence that people have in the COVID-19 preventive behaviors adopted [13]. Under a good safety climate, organizations have specific safety goals as well as strategies and methods for goal implementation. A climate of safety can strengthen employees’ safety values and their recognition of safety as a priority [16]. The research conducted by Akanni et al. [47] also showed that workplace safety climate positively affects self-efficacy, and self-efficacy is the mediator between safety climate and workplace safety behaviors. Therefore, we proposed Hypothesis H1e of the study.

**Hypothesis** **1e (H1e).** *Workplace safety climate will* *positively affect self-efficacy.*

### 2.3. Safety Attitude

Attitude is an important psychological construct, and it can affect and predict behaviors [48]. Safety attitude refers to an individual’s positive and negative viewpoints towards pandemic prevention behaviors. It can be used as a forecasting indicator of dangerous behaviors and affect the probability of incidents [49]. When a person has higher perceived susceptibility, the risk perception of suffering from diseases is higher [33,39]. Park and Oh [50] integrated the Theory of Planned Behavior (TPB) and the health belief model (HBM), and they argued that higher perceived susceptibility and perceived severity reflect the perception of the disease and are important factors in promoting health behaviors. Rimpeekool et al. [51] integrated the Knowledge–Attitude–Behavior model (KAB) and the health belief model, and their findings revealed that the attitude towards using nutrition labels is determined by personal health knowledge and the perception of a healthy diet (perceived susceptibility and perceived severity). From their research outcomes on dietary supplements, Tzeng and Ho [52] pointed out that the perception of health threats will affect the subsequent attitude towards the product when customers’ perceived susceptibility increases. In summary, the risk perception of suffering from the disease or the perception of the health threat will be enhanced when the perceived susceptibility is higher. Therefore, the safety attitude towards pandemic prevention behaviors will also be more positive. We proposed Hypothesis H2a of the study.

**Hypothesis** **2a (H2a).** *Perceived susceptibility will positively* *affect safety attitude.*

The personal evaluation of the consequences of the disease affects pandemic prevention behaviors and attitudes [13]. Previous studies reported that perceived severity affects subsequent safety attitudes and pandemic prevention behaviors [53]. In their research on COVID-19, Park and Oh [50] highlighted that perceived severity is the belief that daily life will be seriously affected when individuals are infected with a disease. The research revealed that perceived severity positively affects the attitude towards pandemic prevention behaviors. Seong and Bae [54] integrated the TPB and HBM to explore preventive behaviors towards COVID-19 among adults. The research found that perceived severity affects prevention behaviors through attitude. In research on the preventive behavior of COVID-19 social distancing, Mai et al. [55] revealed that perceived severity positively affects people’s safety attitudes towards social distancing. Therefore, we proposed Hypothesis H2b of the study.

**Hypothesis** **2b (H2b).** *Perceived severity will positively* *affect safety attitude.*

Perceived benefits refer to pandemic prevention behaviors that can effectively reduce the threat of the disease and other potential advantages [56]. In their research on street food, Choi et al. [57] pointed out that consumers’ perceived benefits positively affect their attitudes. In addition, consumers’ attitudes towards street food play the role of a full mediator between perceived benefits and behavioral intentions. Previous studies showed that individuals hold more positive attitudes towards behaviors when they perceive benefits from them. This means that perceived benefits will generate positive impacts on safety attitudes [58,59]. Moreover, Yasa et al. [60] also indicated that people’s perceived benefits of the continuous use of medical masks significantly affect their attitudes. Therefore, we proposed Hypothesis H2c of the study.

**Hypothesis** **2c (H2c).** *Perceived benefits will positively* *affect safety attitude.*

Perceived barriers are the cost evaluation of the adoption of health behaviors carried out by an individual [39]. The personal cost evaluation of adopting a new measure or behavior affects its acceptance by users [61]. Therefore, the Technology Acceptance Model (TAM) proposes that the perceived ease of use affects the attitude and behavior of acceptance of using new technology [62]. Seong and Bae [54] also revealed that the perceived barriers to COVID-19 preventive behaviors negatively affect safety attitudes. In their research on adults in Iran, Zarei et al. [63] demonstrated that the main perceived barriers to COVID-19 preventive behaviors are a lack of risk perception, the economy, and finance, as well as a lack of protection devices and cultural barriers. Moreover, perceived barriers negatively affect attitudes towards preventive behaviors. Therefore, higher perceived barriers to pandemic prevention measures will reduce positive safety attitudes towards epidemic prevention measures among healthcare workers. Therefore, we proposed Hypothesis H2d of the study.

**Hypothesis** **2d (H2d).** *Perceived barriers will negatively* *affect safety attitude.*

Self-efficacy means an individual’s perception of their capability of successfully performing a behavior [64,65]. In addition, self-efficacy refers to an individual’s belief that he/she has the capability to overcome COVID-19 challenges [10]. An individual’s confidence in performing pandemic prevention behaviors positively affects his or her safety attitude. Previous studies revealed that people with higher self-efficacy have better attitudes and intentions towards health behaviors and believe they are capable of overcoming barriers to implementing a certain health behavior [66,67]. Empirical research conducted by Joveini et al. [68] showed a direct and significant correlation between safety attitude towards COVID-19 and self-efficacy. In their research on dentists in Iran, Zeidi and Zeidi [69] pointed out that there is a significant positive correlation between self-efficacy and attitude. Therefore, we proposed hypothesis H2e of the study.

**Hypothesis** **2e (H2e).** *Self-efficacy will positively* *affect safety attitude.*

### 2.4. Safety Compliance

Safety compliance means that people carry out activities related to safety to maintain workplace safety [19], such as following standard operating procedures and wearing protective equipment. Basahel [70] argued that safety compliance refers to implementing work safely to maintain workplace safety, such as using personal protective equipment or complying with safety rules. Borman and Motowidlo [71] classified performance into task performance and contextual performance. Task performance is the overall expected value of products or services generated in the organization by personal behaviors within a standard period of time. Contextual performance means the overall expected value of maintaining and strengthening mental, social, and organizational work contexts through personal behaviors within a standard period of time [72]. Task performance is regulated by organizations and is related to the core technology and activity of work tasks. Contextual performance is based on the concept of Organizational Citizenship Behavior (OCB). In this study, safety compliance adopts concepts related to task performance and the need to implement core safety tasks by individuals to maintain workplace safety [73].

The concept of safety compliance is similar to cues to action in the HBM. Cues to action are reminders to individuals of the actions they should take to respond to special challenges such as COVID-19 [10]. Cues to action are kinds of cues or triggers that prompt overt actions. The cues can be internal (such as a physical situation) or external (such as interpersonal interaction or media influence) [9]. Experiences directly related to COVID-19 can be regarded as external cues to action [65]. Safety compliance is a protective behavior carried out interpersonally or personally to respond to the direct experience of COVID-19. Therefore, the concept of safety compliance is aligned with the notion of external cues to action.

Because safety attitude is related to employees’ workplace safety beliefs, recognition, and values, it reflects personal opinions on safety policies, procedures, and practices, including personal obligation and personal responsibility for safety [74]. Therefore, personal safety attitude towards pandemic prevention behaviors affects safety compliance. Some previous studies revealed a positive correlation between personal safety attitude and safety performance [74,75,76]. In addition, in their research on the electrical construction industry in Saudi Arabia, Basahel [70] revealed that safety attitude affects safety compliance through safety motivation. Li and Griffin’s [77] research during the COVID-19 pandemic showed that workers’ perceptions of the safety commitment (safety attitude) made by supervisors affects safety behaviors. Therefore, we proposed Hypothesis H3 of the study.

**Hypothesis** **3 (H3).** *Safety attitude will positively* *affect safety compliance.*

### 2.5. Safety Satisfaction

Safety satisfaction is personal satisfaction with organizational pandemic prevention initiatives [78]. When individuals hold positive attitudes towards pandemic prevention behaviors, they will be satisfied with organizational safety policies if organizations adopt proper pandemic prevention initiatives. Previous studies showed that when individuals hold positive attitudes towards organizational policies, they will feel satisfied with the implementation of the policies by their organizations. That is, there is a correlation between patients’ or healthcare workers’ attitudes towards health policies and overall satisfaction [79,80]. The overall satisfaction with medical services from patients or healthcare workers is based on their trust in the medical services and their reaction to the health policies and attitudes of medical institutions [80]. According to the research conducted by Li and Griffin [77] during the COVID-19 pandemic, workers’ perceptions of the safety commitment of supervisors (safety attitude) will affect their work satisfaction. Therefore, we proposed Hypothesis H4 of the study.

**Hypothesis** **4 (H4).** *Safety attitude will positively* *affect safety satisfaction.*

Safety compliance is the personal performance of pandemic prevention behaviors and is a part of task performance proposed by Borman and Motowidlo [71]. It is related to the work tasks and core activities regulated by organizations. When the personal performance of pandemic prevention behaviors is higher, it shows a higher level of implementation of pandemic prevention tasks. It is helpful for the personal recognition of organizational efforts in pandemic prevention. Previous studies also revealed that effective safety initiatives and policy implementation will help in reducing accidents, injuries, and other environmental conditions unfavorable for work [81,82] and further enhance safety satisfaction among employees. In addition, the findings of the research by Rosiek et al. [83] on patients with type 2 diabetes revealed that patients’ health behaviors (proper eating habits, health practices, preventive behaviors, and positive mental attitude) have a significant correlation with satisfaction. Therefore, we proposed Hypothesis H5 of the study.

**Hypothesis** **5 (H5).** *Safety compliance will positively* *affect safety satisfaction.*

## 3. Methods

### 3.1. Research Framework

**Figure 1:**
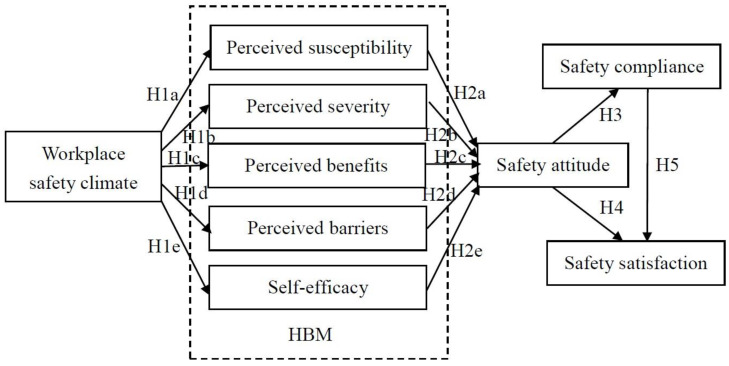
Research framework.

The purpose of this study was to explore factors affecting pandemic prevention behaviors among healthcare workers. The factors include workplace safety climate, health belief factors (perceived susceptibility, perceived severity, perceived benefits, perceived barriers, and self-efficacy), and safety attitude. The research framework is shown in Figure 1.

### 3.2. Measurement and Data Collection

The design of the questionnaires in this study referred to previous domestic and overseas empirical research and the environmental context of medical institutions during the COVID-19 pandemic to develop a structural questionnaire. The measurement questionnaire included demographic variables, such as gender, age, marital status, educational background, monthly salary, job position, seniority, and department, as well as nine subscales, including workplace safety climate, perceived susceptibility, perceived severity, perceived benefits, perceived barriers, self-efficacy, safety attitude, safety behaviors (compliance), and safety satisfaction, adopting a 5-point Likert scale. Workplace safety climate included three questions that were modified from research by Neal and Griffin [84] as well as Liu and Lu [85]. The five constructs in the health belief model, including perceived susceptibility (3 questions), perceived severity (2 questions), perceived benefits (3 questions), perceived barriers (3 questions), and self-efficacy (3 questions), were modified from Huang et al. [86] and Ban and Kim [28]. Safety attitude consisted of three questions that were modified from Ajzen [87] and Aschwanden et al. [88]. Safety compliance contained three questions that were modified from Neal and Griffin [84] as well as Huang et al. [20]. Safety satisfaction included six questions that were modified from Cheah et al. [78] and Huang et al. [20]. The data of the study were collected from March to August 2021, and the research subjects were healthcare workers of a famous medical institution in Taipei City.

This study conducted a web survey through SurveyCake. The response rate of valid questionnaires was 85%, with 273 valid questionnaires in total. In terms of the descriptive statistics of effective samples, there were more female respondents (89.01%) than male respondents (10.99%). In terms of age profiles, the group of participants between 40 and 49 years old was the largest (33.33%), followed by the group of 50–59-year-old participants (23.44%). In terms of marital status, respondents with a married status were the majority (52.38%). As for educational level, most of the respondents had university degrees (77.66%). The majority had a monthly salary between TWD 40,001 and TWD 60,000 (60.07%), while job positions were mainly non-management levels (81.68%), and a seniority of more than 21 years was the most common (31.50%). In terms of departments, the nursing department was predominant (40.29%), followed by hospital administration (medical business, general affairs, operation, human resource, information, engineering, finance, and administrators), which accounted for 34.43%. In terms of religion, most of them had no religion (43.96%). All respondents’ demographic information is shown in Table 1.

## 4. Results

### 4.1. Reliability and Validity Analysis

**Figure 2:**
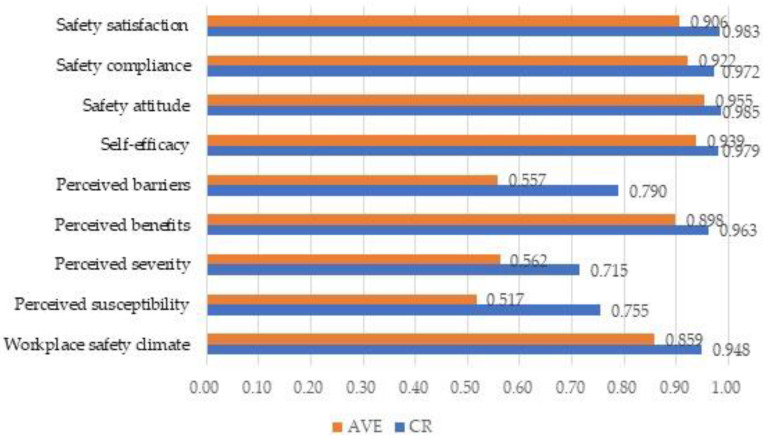
Graph of confirmatory factor analysis.

In terms of reliability and validity analysis, the study used a confirmatory factor analysis (CFA) before conducting a causal analysis. First, reliability was measured by a statistical coefficient, Cronbach’s α, to test the level of internal consistency between the measured variables. Good consistency is usually represented by Cronbach’s α value above 0.7 [89]. The test results showed that Cronbach’s α values for workplace safety climate, perceived susceptibility, perceived severity, perceived benefits, perceived barriers, self-efficacy, safety attitude, safety compliance, and safety satisfaction were 0.906, 0.672, 0.640, 0.922, 0.691, 0.933, 0.947, 0.926, and 0.975, respectively. Cronbach’s α values for all dimensions were close to or over 0.7, which indicated that the internal consistency achieved an acceptable level. In terms of convergent validity, the confirmatory factor analysis in Table 2 and Figure 2 showed that the composite reliability (CR) of all questions is over or close to 0.6. This represents the acceptable internal consistency of the research constructs [90]. In addition, the average variance extracted (AVE) values were all above 0.5, which is an acceptable level. These results showed that each construct had suitable convergent validity.

This study used correlation coefficients between two constructs to measure discriminant validity based on whether the coefficient is smaller than the square root of the AVE value for each construct [90]. As shown in Table 3, each correlation coefficient was less than the square root of the AVE value of each construct. Therefore, there was good discriminant validity in each construct.

### 4.2. Outcome of Hypothesis Testing

**Figure 3:**
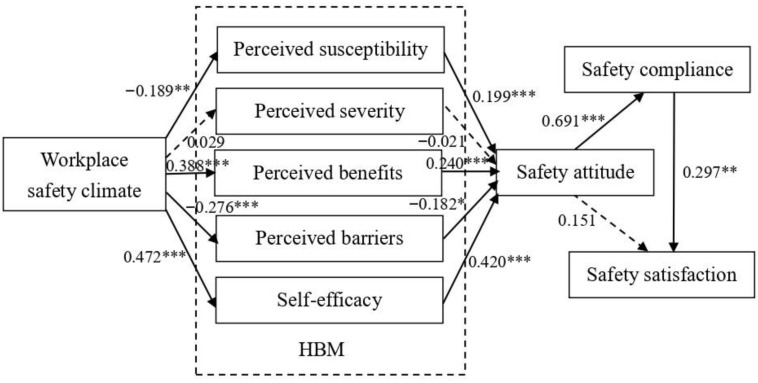
Results of structural model analysis. Note: * *p* < 0.05; ** *p* < 0.01; *** *p* < 0.001.

This study used AMOS software for causal analysis to test whether the research hypotheses achieve the level of significance. The overall model goodness-of-fit indices were χ^2^/df = 2.590, Goodness-of-Fit Index (GFI) = 0.833, Non-normed Fit Index (NFI) = 0.883, Comparative Fit Index (CFI) = 0.924, Incremental Fit Index (IFI) = 0.925, and Root-Mean-Square Residual (RMR) = 0.093. The results of the verified research hypotheses and structural model analysis are shown in Table 4 and Figure 3.

Firstly, workplace safety climate had significant negative effects on perceived susceptibility and perceived barriers. The path coefficients were −0.189 and −0.276, respectively, and the *p* values were less than 0.01 and 0.001. This revealed that when the workplace safety climate was higher, the perceived susceptibility and perceived barriers of healthcare workers would be lower. Therefore, H1a and H1d of the study were supported. However, workplace safety climate did not have a significant positive effect on perceived severity. The path coefficient was not significant; therefore, H1b was not supported. In addition, workplace safety climate had significant positive effects on perceived benefits and self-efficacy. The path coefficients were 0.388 and 0.472, respectively, and the *p* values were all less than 0.001. This revealed that when the workplace safety climate was higher, the perceived benefits and self-efficacy of healthcare workers would also be higher. Therefore, H1c and H1e of the study were supported.

Secondly, Table 2 reveals that perceived susceptibility, perceived benefits, and self-efficacy all had significant positive effects on safety attitude. The path coefficients were 0.199, 0.240, and 0.420, respectively, while the *p* values were all less than 0.001. Therefore, H2a, H2c, and H2e of this study were all supported. Moreover, perceived severity did not have a significant positive effect on safety attitude. The path coefficient was not significant; therefore, H2b was not supported. Perceived barriers had a significant negative effect on safety attitude. The path coefficient was −0.182, and the *p* value was less than 0.05. This revealed that when perceived barriers were higher, safety attitude would be lower. Therefore, H2d of the study was supported.

Finally, safety attitude had a significant positive effect on safety compliance. The path coefficient was 0.691, and the *p* value was less than 0.001. This revealed that when safety attitude was higher, safety compliance would also be higher. Therefore, H3 of this study was supported. In addition, safety attitude did not have a significant positive effect on safety satisfaction. The path coefficient was not significant. Therefore, H4 of the study was not supported. Lastly, safety compliance had a significant positive effect on safety satisfaction. The path coefficient was 0.297, and the *p* value was less than 0.001. This revealed that when safety compliance was higher, safety satisfaction would also be higher. Therefore, H5 of the study was supported.

## 5. Discussion and Conclusions

The purpose of this study was to integrate workplace safety climate and the HBM to explore COVID-19 pandemic prevention behaviors among healthcare workers. With the conceptual research framework established through a literature review, it was then verified with an empirical sample that was drawn from healthcare workers of a famous medical institution in Taipei.

First of all, in terms of the correlation between workplace safety climate and the HBM, workplace safety climate had a significant positive effect on perceived susceptibility. That is, when employees possess a greater consensus on safety policies, procedures, and practices implemented in the medical institution, it will be more helpful in enhancing the awareness of being infected with the disease among healthcare workers. Therefore, the management levels of the medical institution should strengthen the transparency of pandemic prevention policies and initiatives implemented in the hospital, promote the understanding of pandemic prevention behaviors among healthcare workers, and highlight the importance of pandemic prevention behaviors to enhance the awareness of pandemic prevention among healthcare workers. However, workplace safety climate did not have a positive effect on perceived severity. This may be because there were not too many confirmed cases in Taiwan at that moment; therefore, many people (including healthcare workers) did not think the pandemic was severe. A study on adults in Taiwan receiving COVID-19 vaccines and carrying out protective actions showed that 63.5% of people in Taiwan still felt that the situation of COVID-19 was not serious [3]. In addition, the risk perception of being infected with COVID-19 was not only from their workplace but also from other information sources. That is, the level of trust in COVID-19 information sources is associated with perceived severity (the perception of risk) [91]. Therefore, unexpected results appeared in this study. In addition, workplace safety climate had a positive effect on perceived benefits. That is, a safe working environment can help employees feel at ease and further enhance employees’ perceptions of the benefits of pandemic preventive behaviors.

The findings of this research showed that workplace safety climate had a significant negative effect on perceived barriers. The barriers to COVID-19 pandemic preventive behaviors among healthcare workers in hospitals include failure to provide proper personal protective equipment, insufficient supportive medication, and a lack of proper ventilation equipment [45]. This revealed that when hospitals focus more on pandemic prevention, it reduces barriers to the prevention behaviors of healthcare workers. They are more unlikely to regard the additional work caused by pandemic prevention as a barrier to their daily work. In addition, when the pandemic prevention actions carried out by hospitals are safer, healthcare workers will be more confident to overcome obstacles to the implementation of pandemic prevention. According to the results of this research, workplace safety climate had a significant positive effect on self-efficacy. The organization should build policies for complete and comprehensive pandemic prevention first, which in turn can promote specific measures, strategies, and methods for the implementation of pandemic prevention and can also strengthen employees’ level of confidence in pandemic prevention. From the above, the safety solutions, policies, and practical actions conducted in hospitals are helpful for enhancing healthcare workers’ health beliefs. For example, hospital executives can improve healthcare workers’ health beliefs through an epidemic prevention awareness campaign (improve the perceived benefits of the preventive measures and increase the perceived severity of COVID-19) [92]. They can also support and strengthen the pandemic preventive measures of medical institutions, including proper personal protective equipment, sufficient supportive medication, and proper ventilation equipment [45]. In addition, healthcare workers can also be provided with education programs on pandemic prevention demonstrating that they need to perform preventive actions more effectively [54]. Therefore, making pandemic prevention initiatives concrete and helping healthcare workers comply with clear pandemic prevention policies are key points that the management levels of hospitals have to focus on.

Regarding the correlation between the HBM and safety attitude, previous studies pointed out that safety attitude refers to individuals’ evaluation and positive and negative opinions on pandemic prevention measures [49,75]. The results of this study showed that individuals’ opinions on the probability of suffering from the disease, risks, external messages of pandemic prevention, and their level of confidence will all affect personal safety attitudes and behaviors towards pandemic prevention [13,39,67]. Therefore, for management levels, strengthening healthcare workers’ health beliefs, including the risk of being infected with the disease, and reducing barriers to pandemic prevention behaviors are helpful for enhancing safety attitudes. Past studies have pointed out that positive health beliefs can reduce the risk of disease [93], increase participation in preventive behaviors [94], and create a more positive view of epidemic preventive behaviors. However, perceived severity did not have a positive effect on safety attitude. This may be because the feelings towards safety attitudes among healthcare workers are different from those of the general public. The development of a safety attitude comes from the long-term education of medical professionals as well as daily practices and training provided to healthcare workers in hospitals, but not directly from personal perception of the seriousness of being infected. In addition, past studies have pointed out that whether perceived severity affects attitude depends on the chance of infection. If the infection is detected early and treated properly, the perceived severity is not high [95]. Therefore, there was no direct impact. According to the present findings, the HBM is a suitable conceptual framework that can be used to promote COVID-19 pandemic preventive behaviors among healthcare workers. Health institutions and hospitals should focus on ways to reinforce the perception of threat susceptibility, to improve the benefits of practicing preventive behaviors (perceived benefits), to overcome the efforts and costs of implementing preventive initiatives (perceived barriers), and to provide the necessary resources and support for taking preventive actions (self-efficacy) [10,29,65].

In addition, in terms of safety attitude and safety compliance, this study proved that there was a positive correlation between personal safety attitude and safety compliance [74,75,76]. Therefore, strengthening personal positive attitudes towards pandemic prevention behaviors will help in enhancing the overall safety performance in hospitals and improve personal safety compliance.

Lastly, in terms of safety attitude and safety satisfaction, this study did not show a significant impact. The possible reasons for this are that a personal positive attitude and the evaluation of pandemic prevention initiatives fail to fully reflect personal satisfaction with pandemic prevention initiatives implemented in hospitals. This showed that even though hospitals have relevant pandemic prevention initiatives in place, individuals may still have different opinions on the specific measures and behaviors for pandemic prevention. A previous study indicated that employees’ perceptions of change (the frequency of change, the planning involved in change, and transformational change) will affect their attitudes (uncertainty), which in turn influence their job satisfaction [96]. Li and Griffin [77] also showed that psychological uncertainty plays a mediating role between the experience of COVID-19 and satisfaction. In addition, satisfaction comes from expectations, and the higher the expectations, the harder it is to satisfy them [97]. Furthermore, the perceived managerial safety commitment positively affected job satisfaction during the pandemic [77]. Therefore, higher safety satisfaction with pandemic preventive measures depends not only on positive attitudes and evaluations but also on their expectations for medical institutions and managerial safety commitment. However, safety compliance positively affected safety satisfaction. Therefore, for management levels in hospitals, compliance with safety criteria by healthcare workers will help in enhancing healthcare workers’ safety satisfaction with hospitals. This is why strengthening safety compliance behaviors among healthcare workers is one of the key factors in enhancing satisfaction with pandemic prevention. Past studies have also pointed out that safety compliance guidelines can improve the safety of medical behaviors [98] and, therefore, can improve safety satisfaction. Thus, information and knowledge about prevention, mitigation, and operation initiatives and guidelines should be disseminated to all healthcare workers in the hospital via various online communication platforms or social media [77].

### 5.1. Implications

In terms of implications for management, the above results of this research showed that workplace safety climate positively affected the health beliefs of healthcare workers. Therefore, if hospitals are able to continue strengthening their safety policies and procedures in the hospital, it will help promote employees’ awareness of pandemic prevention. As a result, it is suggested that management levels in hospitals continue promoting relevant pandemic prevention solutions and policies as well as strengthening the mechanism of controlling infection. Moreover, all daily work must meet safety regulations. In addition, rolling-wave planning and control measures should be implemented according to pandemic announcements or press releases published by the Taiwan Center for Disease Control [2] every day. Hospitals should provide sufficient and good-quality personal protective equipment and resources to employees as well as ensure employees are all vaccinated to reduce the risk of cross-infection and enhance the safety level of the hospital. Hospitals should also establish good policies, incentives, and regulations to make sure employees feel they are protected [99]. Moreover, if management levels present their commitment to pandemic prevention policy implementation and give priority to safety concerns, employees will reciprocate by complying with pandemic prevention behaviors [47].

This study also proved that among the factors of the health belief model, perceived susceptibility, perceived benefits, and self-efficacy had significant positive effects on safety attitude, while perceived barriers had a significant negative effect on safety attitude. As a whole, the research outcomes were consistent with the results from previous studies [11,14]. Therefore, employees in hospitals believe they are facing a severe threat of infectious disease and are more likely to perform preventive behaviors. Based on this, the promotion of relevant educational programs, including COVID-19 pandemic prevention guidelines and on-the-job training, is extremely important for frontline workers. Moreover, management levels in hospitals should widely circulate medical and health information related to pandemic prevention measures, such as vaccination, through social media, electronic bulletins, or hospital websites. Doing so will urge health workers in hospitals to more actively participate in pandemic prevention. As for strengthening self-efficacy, empowerment training and practices on healthcare workers’ pandemic prevention capabilities will enhance the self-efficacy of pandemic prevention among employees. Lastly, reducing potential obstacles to pandemic prevention to the minimum or removing obstacles, such as installing more hand-washing equipment, providing rubbing alcohol for hand sanitizers, and providing sufficient resources such as pandemic prevention supplies, will help in the implementation of pandemic prevention behaviors.

Furthermore, healthcare workers’ attitudes towards pandemic prevention positively affected safety compliance behaviors. That is, personal positive opinions on pandemic prevention affect the subsequent performance of pandemic prevention behaviors. Therefore, strengthening healthcare workers’ awareness of pandemic prevention has become the key point of pandemic prevention initiatives. In particular, COVID-19 has been spreading for a while now. Management levels in hospitals must pay special attention to whether the awareness of pandemic prevention among healthcare workers has started to relax. As the frontliners of pandemic prevention, the necessity of pandemic prevention measures and relevant practices must be promoted regularly, including guidelines on pandemic prevention behaviors, guidelines on infection control measures, the disclosure of statistics on pandemic prevention capacities in hospitals, and even mental counseling and stress reduction for pandemic prevention. These must be continued, and their performance should be tracked by management levels in hospitals. Because COVID-19 might cause physical and mental fatigue, increase workload, and increase mental stress in some healthcare workers, hospitals must establish care mechanisms and supportive measures related to mental therapy or mental counseling, as well as flexible management systems and measures to respond to the dynamic adjustment of pandemic prevention policies published by the central government at any time and further ensure healthcare workers continue holding positive attitudes towards pandemic prevention behaviors. By doing so, the efforts towards pandemic prevention safety in hospitals can operate sustainably.

### 5.2. Limitations and Future Research

First, due to limitations on budget, time, and manpower, this study only used healthcare workers in one of the famous medical institutions in Taipei City as the subjects. There was no further evidence-based research in hospitals or relevant medical institutions (such as long-term care institutions) in other areas. In the future, it is suggested to carry out in-depth research in medical institutions with different characteristics. In addition, this study only used cross-sectional data as empirical evidence. Therefore, we were unable to observe changes in impacts on the variables of safety compliance and safety satisfaction caused by different pandemic prevention regulations and measures adopted by hospitals along with changes in the pandemic. It is suggested that researchers in the future collect empirical evidence at different points of time for discussion if time and manpower allow. In the end, the results of this evidence-based research showed significant relationships among workplace safety climate, health beliefs, safety attitude, safety compliance, and safety satisfaction, but no significant relationship between safety attitude and safety satisfaction. Whether it was interfered with by other personal variables is something that future researchers can explore in depth.

## Figures and Tables

**Table 1:** Respondents’ demographic information.

Variable	Category	Frequency	Percentage (%)
Gender	Male	30	10.99%
	Female	243	89.01%
Age	20–29	47	17.22%
	30–39	56	20.51%
	40–49	91	33.33%
	50–59	64	23.44%
	60–69	14	5.13%
	>70	1	0.37%
Marital status	Married	143	52.38%
	Not married	118	43.22%
	Others	12	4.40%
Educational level	High school	6	2.20%
	University	212	77.66%
	Graduate school	55	20.14%
Monthly salary (TWD)	<20,000	5	1.83%
	20,001–40,000	50	18.32%
	40,001–60,000	164	60.07%
	60,001–80,000	42	15.38%
	80,001–100,000	3	1.10%
	>100,000	9	3.30%
Job position	Management	50	18.32%
	Non-management	223	81.68%
Seniority (Year)	1–5	69	25.28%
	6–10	53	19.41%
	11–15	26	9.52%
	16–20	39	14.29%
	>20	86	31.50%
Department	Hospital administration	94	34.43%
	Physician	9	3.30%
	Nurse	110	40.29%
	Others	60	21.98%
Religion	No religion	120	43.96%
	Christianity	51	18.68%
	Buddhism	53	19.41%
	Taoism	37	13.55%
	Catholic	2	0.73%
	Others	10	3.67%

**Table 2:** Results of confirmatory factor analysis.

Construct	Question Item	Factor Loading	CR	AVE
Workplace safety climate	The hospital emphasizes pandemic prevention initiatives very much.	0.900 ***	0.948	0.859
	The hospital puts the safety of healthcare workers in the highest priority.	0.871 ***		
	The hospital believes pandemic prevention initiatives are important.	0.861 ***		
Perceived susceptibility	I feel I have a very high risk of suffering from COVID-19.	0.601 ***	0.755	0.517
	I am worried about being infected with COVID-19.	0.817 ***		
	I feel very painful when I am sick.	0.514 ***		
Perceived severity	If I have COVID-19, it will totally change my life. I am afraid of being infected with COVID-19.	0.584 *** 0.806 ***	0.715	0.562
Perceived benefits	Pandemic prevention initiatives help me reduce the risk of suffering from COVID-19.	0.850 ***	0.963	0.898
	Pandemic prevention initiatives make me feel at ease.	0.930 ***		
	Pandemic prevention initiatives protect my health.	0.903 ***		
Perceived barriers	I usually forget to perform pandemic prevention initiatives.	0.661 ***	0.790	0.557
	I have other things that are more important than pandemic prevention initiatives to deal with.	0.665 ***		
	Pandemic prevention initiatives are barriers for daily work.	0.642 ***		
Self-efficacy	I know how to perform pandemic prevention initiatives.	0.925 ***	0.979	0.939
	I can perform pandemic prevention initiatives correctly.	0.949 ***		
	I believe I can overcome barriers and perform pandemic prevention initiatives.	0.851 ***		
Safety attitude	I have a positive attitude to perform pandemic prevention initiatives.	0.954 ***	0.985	0.955
	I believe it is wise to perform pandemic prevention initiatives.	0.928 ***		
	I think it is necessary to perform pandemic prevention initiatives.	0.900 ***		
Safety compliance	I use necessary pandemic prevention equipment to carry out my work.	0.939 ***	0.972	0.922
	I use correct pandemic prevention procedures to carry out my work.	0.944 ***		
	When I carry out my work, I will make sure the highest level of pandemic prevention safety is achieved.	0.831 ***		
Safety satisfaction	I am very satisfied with the pandemic prevention committee in the hospital.	0.902 ***	0.983	0.906
	I am very satisfied with the protective initiatives performed in the hospital.	0.922 ***		
	I am very satisfied with the audit and inspection carried out in the hospital. I am very satisfied with the reporting procedures for incidents happened in the hospital.	0.941 *** 0.953 ***		
	I am very satisfied with the handling procedures for incidents carried out in the hospital.	0.940 ***		
	In summary, I am very satisfied with the protective mechanism for COVID-19 used in the hospital.	0.935 ***		

χ^2^ = 760.294, df = 341; χ^2^/df = 2.230, GFI = 0.841, TLI = 0.933, CFI = 0.943, RMSEA = 0.067, *** *p* < 0.001.

**Table 3:** Results of discriminant validity analysis.

Construct	1	2	3	4	5	6	7	8	9
Workplace safety climate	0.927								
Perceived susceptibility	−0.192 **	0.719							
Perceived severity	0.042	0.459 **	0.750						
Perceived benefits	0.362 **	−0.012	0.170 **	0.948					
Perceived barriers	−0.180 **	0.072	0.107	−0.202 **	0.746				
Self-efficacy	0.456 **	0.065	0.006	0.425 **	−0.456 **	0.969			
Safety attitude	0.466 **	0.199 **	0.140 **	0.404 **	−0.344 **	0.600 **	0.977		
Safety compliance	0.521 **	0.109	0.175 **	0.495 **	−0.369 **	0.639 **	0.677 **	0.960	
Safety satisfaction	0.746 **	−0.192 **	0.028	0.367 **	−0.142*	0.398 **	0.351 **	0.418 **	0.952

Note: ** *p* < 0.01; the diagonal is the square root of the AVE value of each construct, while the non-diagonal values are correlation coefficients between two constructs.

**Table 4:** Research hypotheses and test results.

Hypothesis	Path	Standardized Path Coefficient	Result of the Hypothesis
H1a	Workplace safety climate → Perceived susceptibility	−0.189 **	Supported
H1b	Workplace safety climate → Perceived severity	0.029	Not supported
H1c	Workplace safety climate → Perceived benefits	0.388 ***	Supported
H1d	Workplace safety climate → Perceived barriers	−0.276 ***	Supported
H1e	Workplace safety climate → Self-efficacy	0.472 ***	Supported
H2a	Perceived susceptibility → Safety attitude	0.199 ***	Supported
H2b	Perceived severity → Safety attitude	−0.021	Not supported
H2c	Perceived benefits → Safety attitude	0.240 ***	Supported
H2d	Perceived barriers → Safety attitude	−0.182 *	Supported
H2e	Self-efficacy → Safety attitude	0.420 ***	Supported
H3	Safety attitude → Safety compliance	0.691 ***	Supported
H4	Safety attitude → Safety satisfaction	0.151	Not supported
H5	Safety compliance → Safety satisfaction	0.297 **	Supported

Note: * *p* < 0.05; ** *p* < 0.01; *** *p* < 0.001.

## Data Availability

Data generated during the study. The data is unavailable due to privacy or ethical restrictions.
